# Supplementary material for: EnzML: multi-label prediction of enzyme classes using InterPro signatures
Source: BMC Bioinformatics. 2012 Apr 25;13:61. doi: 10.1186/1471-2105-13-61 (PMC3483700; doi:10.1186/1471-2105-13-61)
Supplement: Addtional file 5 — The Java code to format the data files, evaluate and predict. The file enzml_java_code.tar.gz contains the Java code used to format database data to ARFF and XML formats, to execute cross and train-test (jackknife) evaluations and to record evaluation results to database. More information is included in the readme.txt file and the Javadoc files. The code can be used with a MySQL database. To use a different database software, other JDBC drivers might be required. [file 1471-2105-13-61-S5.gz › java_code/utils/doc/uk/ac/ed/inf/utils/stats/StatUtils.html]

StatUtils


---


|  |  |  |
| --- | --- | --- |
| **Overview** \| **Package** \| **Class** \| **Use** \| **Tree** \| **Deprecated** \| **Index** \| **Help** | |  |
| **PREV CLASS**   **NEXT CLASS** | **FRAMES**    **NO FRAMES**     **All Classes** |  |
| SUMMARY: NESTED \| FIELD \| CONSTR \| METHOD | DETAIL: FIELD \| CONSTR \| METHOD |  |


---


## uk.ac.ed.inf.utils.stats Class StatUtils

```
java.lang.Object
  uk.ac.ed.inf.utils.stats.StatUtils
```

---

``` public class StatUtils extends java.lang.Object ```

Utilities to print simple histograms, percent of frequency etc

**Version:**
:   19 Jul 2008

**Author:**
:   Luna De Ferrari luna.deferrari-at-ed.ac.uk

---

**Constructor Summary**

`StatUtils()`


| **Method Summary** | |
| --- | --- |
| `static java.util.TreeMap<java.lang.Integer,java.lang.Integer>` | `arrayFrequencies(java.lang.Integer[] numbers)`             Records the freqencies with which the integers appear in the array |
| `static java.util.TreeMap<java.lang.Integer,java.lang.Double>` | `fromFrequencyToPercOfFrequency(java.util.TreeMap<java.lang.Integer,java.lang.Integer> frequencies)`             Converts a frequency from number of events to percentage over the total number of exctractions |
| `static int` | `getRandomInt(int upperLimitPlusOne)`             Problem: generates a new Random at each call... |
| `static java.lang.String` | `histogram(java.util.TreeMap<java.lang.Integer,java.lang.Integer> frequencies)`             Returns a frequency histogram |
| `static java.lang.String` | `histogramBar(java.lang.Integer integer, java.lang.Integer frequency)`             Returns an histogram bar |
| `static java.util.TreeMap<java.lang.Integer,java.lang.Integer>` | `paretoFrequencies(Pareto pareto, int numberOfExtractions)`             Extracts numbers from a pareto distribution for the given number of times and records the freqencies with which the integers have been extracted |
| `static java.util.TreeMap<java.lang.Integer,java.lang.Integer>` | `vectorFrequencies(java.util.Vector<java.lang.Integer> vector)`             Records the freqencies with which the integers appear in the array |

**Methods inherited from class java.lang.Object**

`equals, getClass, hashCode, notify, notifyAll, toString, wait, wait, wait`

**Constructor Detail**

### StatUtils

```
public StatUtils()
```


**Method Detail**

### arrayFrequencies

```
public static java.util.TreeMap<java.lang.Integer,java.lang.Integer> arrayFrequencies(java.lang.Integer[] numbers)
```

:   Records the freqencies with which the integers appear in the array

    :   **Returns:**: a map containing an integer as key and a frequency as value (frequency = number of times the integer has been extracted)

---


### fromFrequencyToPercOfFrequency

```
public static java.util.TreeMap<java.lang.Integer,java.lang.Double> fromFrequencyToPercOfFrequency(java.util.TreeMap<java.lang.Integer,java.lang.Integer> frequencies)
```

:   Converts a frequency from number of events to percentage over the total
    number of exctractions

    :   **Parameters:**: `frequencies` - the map of each number and the number of times it was extracted **Returns:**: the map with each integer as key and the percentage of times it was extracted over the total number of extractions

---


### getRandomInt

```
public static int getRandomInt(int upperLimitPlusOne)
```

:   Problem: generates a new Random at each call...

    :   **Parameters:**: `upperLimitPlusOne` - **Returns:**

---


### histogram

```
public static java.lang.String histogram(java.util.TreeMap<java.lang.Integer,java.lang.Integer> frequencies)
```

:   Returns a frequency histogram

    :   **Parameters:**: `frequencies` - a map containing an integer as key and a frequency as value (frequency = number of times the integer has been extracted)

---


### histogramBar

```
public static java.lang.String histogramBar(java.lang.Integer integer,
                                            java.lang.Integer frequency)
```

:   Returns an histogram bar

    :   **Parameters:**: `integer` -: `frequency` - **Returns:**: an histogram bar

---


### paretoFrequencies

```
public static java.util.TreeMap<java.lang.Integer,java.lang.Integer> paretoFrequencies(Pareto pareto,
                                                                                       int numberOfExtractions)
```

:   Extracts numbers from a pareto distribution for the given number of times
    and records the freqencies with which the integers have been extracted

    :   **Parameters:**: `numberOfExtractions` - the number of integer extractions from a a pareto distribution **Returns:**: a map containing an integer as key and a frequency as value (frequency = number of times the integer has been extracted)

---


### vectorFrequencies

```
public static java.util.TreeMap<java.lang.Integer,java.lang.Integer> vectorFrequencies(java.util.Vector<java.lang.Integer> vector)
```

:   Records the freqencies with which the integers appear in the array

    :   **Returns:**: a map containing an integer as key and a frequency as value (frequency = number of times the integer has been extracted)


---


|  |  |  |
| --- | --- | --- |
| **Overview** \| **Package** \| **Class** \| **Use** \| **Tree** \| **Deprecated** \| **Index** \| **Help** | |  |
| **PREV CLASS**   **NEXT CLASS** | **FRAMES**    **NO FRAMES**     **All Classes** |  |
| SUMMARY: NESTED \| FIELD \| CONSTR \| METHOD | DETAIL: FIELD \| CONSTR \| METHOD |  |


---
